# A Rare Case of Extrahepatic Portal Venous Obstruction in a Nine-Year-Old Female and Its Management: A Case Report

**DOI:** 10.7759/cureus.32150

**Published:** 2022-12-03

**Authors:** Uduak A Udo, Tulika Garg, Zainab Talal O Omar, Etaluka Blanche Mungu, Sanathan Aiyadurai, Idoroeyin S Una, Goodness C Sunday, Omolola Ajekigbe, Hassan A Chaudhry, Aadil Khan

**Affiliations:** 1 Radiology, Imaging for Women, Kansas City, USA; 2 Medicine, Government Medical College & Hospital, Chandigarh, IND; 3 Pediatrics, Dubai Medical College for Girls, Dubai, ARE; 4 Preventive Medicine, Gerald Family Care, Washington DC, USA; 5 Internal Medicine, Caribbean Medical University, Willemstad, CUW; 6 Pediatrics, Afe Babalola University, Ado-Ekiti, NGA; 7 Internal Medicine, Ebonyi State University College of Health Sciences, Abakaliki, NGA; 8 Pediatrics, Ladoke Akintola University of Technology, Ogbomoso, NGA; 9 Biological Sciences, Temple University, Philadelphia, USA; 10 Medical School, Medical University of Lublin, Lublin, POL; 11 Interdisciplinary Medicine, Independent Research Scholar, Philadelphia, USA; 12 Internal Medicine, Lala Lajpat Rai Hospital, Kanpur, IND

**Keywords:** portal biliopathy, schistosomiasis, transjugular intrahepatic portosystemic shunt (tips), extrahepatic portal vein obstruction (ehpvo), endotherapy

## Abstract

Extrahepatic portal vein obstruction (EHPVO) is a kind of liver vascular disease that causes structural abnormalities in the portal veins, including cavernomatous metamorphosis and obstruction. It is the most common cause of esophageal varices-related hematemesis in youngsters. Significant risk factors include congenital abnormalities, dehydration, sepsis, trauma, hypercoagulable conditions, and multiple transfusions. Acute extrahepatic portal vein blockage is often ignored because patients are usually asymptomatic. Subacute and chronic stages can cause symptoms including splenomegaly and hematemesis without hepatic decompensation. Imaging studies aid in the diagnosis; Doppler imaging is added to ultrasonography to visualize portal vein blood flow. MRI and CT scans are used to visualize portal vein blockage. Prevention of acute bleeding is the cornerstone in the management. Studies have shown that transhepatic thrombolysis is the preferred choice to avoid systemic side effects. Transjugular intrahepatic portosystemic shunt (TIPS) treats extrahepatic portal venous thrombosis and is typically followed by conservative variceal hemorrhage treatment. Liver transplantation is performed when other management measures fail. Here, we present a rare case of EHPVO in a nine-year-old female who was lost to follow-up for a long time and later showed signs of portal biliopathy and non-visualization of a surgically created splenorenal shunt. Re-shunting was performed after detailed conservative management, and the patient responded well to the treatment given.

## Introduction

Extrahepatic portal vein obstruction (EHPVO) is a form of vascular disease in the liver that leads to structural changes in the portal veins, including cavernomatous transformation and obstruction. It may or may not involve the intrahepatic portal veins. EHPVO is common among the Asian population. It is a common cause of portal hypertension in children and the major cause of hematemesis in the pediatric population due to esophageal varices. It differs from portal vein obstruction due to neoplasms, chronic liver disease, and post-surgical complications [1].

Predisposing factors include congenital anomalies, dehydration, sepsis, trauma, hypercoagulable states, and multiple transfusions. The clinical presentation consists of splenomegaly and painless hematemesis without hepatic decompensation. Schistosomiasis causes perisinusoidal portal obstruction but is similar to EHPVO [2]. Laboratory investigation includes liver function tests that show mild liver enzyme elevation in the absence of chronic liver disease or cirrhosis. EHPVO can be easily missed during the acute phase because patients are usually asymptomatic. Subacute and chronic stages typically cause the clinical manifestations of the disease. Imaging studies are valuable tools for diagnosing the disease. Ultrasonography is the first line of imaging, with the addition of Doppler imaging to visualize the blood flow in the portal vein. MRI and CT scans are the next steps to further visualize the portal vein obstruction. Histopathological findings of liver biopsy do not show significant findings except apoptotic changes in the non-perfused areas with increased mitotic activity in the remaining regions [3].

The treatment goal is to prevent episodes of acute bleeding. Conservative management includes thrombolysis, preferably through the transhepatic route, to avoid systemic side effects. Shunt surgery is preferred for treating variceal bleeding after conservative treatment fails. Transjugular intrahepatic portosystemic shunt (TIPS) is the treatment modality for extrahepatic portal venous thrombosis, and liver transplantation is the last resort when all other treatment options fail [4].

## Case presentation

A nine-year-old female presented to the surgical outpatient department with chief complaints of abdominal pain and vomiting on and off for four years. A history of intermittent melena and hematemesis was documented as well. Her past medical history was unremarkable, with an up-to-date vaccination history. She was afebrile with a heart rate of 60 beats/minute, a respiratory rate of 15 breaths/minute, a blood pressure of 100/60, and oxygen saturation of 98% on room air. On physical examination, the patient was vitally stable and had a soft, non-tender abdomen with significant splenomegaly up to 11 cm below the left costal margin. Her higher mental functions were normal. The rest of the systemic examination was unremarkable. The patient was clinically stabilized via volume resuscitation.

On further evaluation, a hepatic vascular ultrasound study was done, which revealed a mildly enlarged liver, measuring 11.7 cm with normal echotexture and mild central intrahepatic biliary dilation. Splenomegaly with a length of 13 cm was also revealed. In addition, the portal vein and the intrahepatic branches were replaced by multiple collateral channels like splenic hilar and periportal veins. All these impressions were suggestive of EHPVO with portal cavernoma and hypersplenism. The patient was managed with volume resuscitation, proton pump inhibitor (PPI) infusion, and terlipressin. After three days, the patient developed hematemesis. Upper gastrointestinal (GI) endoscopy showed upper GI bleeding due to esophageal varices, owing to which the patient underwent endoscopic variceal band ligation and managed with volume resuscitation, PPI, and vasoconstrictors. However, the patient did not respond well to the treatment given on subsequent follow-ups. The patient was admitted to the hospital, further evaluated, and planned for splenectomy and splenorenal shunt surgery, for which consent was taken from the parents after a detailed discussion related to patient care, description of the procedure, and future management. Before the splenectomy, she was commenced on antibiotics prophylactically and was immunized with recommended vaccines as protocol. The postoperative course was uneventful and postoperative imaging showed a partially recanalized main portal vein with few collaterals at the hilum; the splenorenal shunt was patent.

**Figure 1:**
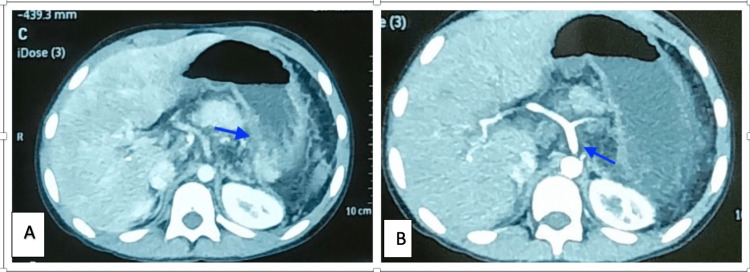
Post splenectomy imaging: (A) splenorenal shunt was not visualized and (B) portal biliopathy was seen

She was discharged and was planned for evaluation after every six weeks. However, she was not compliant with regular checkups, and the patient came one year later for a follow-up. USG findings were suggestive of intrahepatic biliary radicle dilation (IHBRD) with peri gallbladder collaterals. For more evident findings, a triple-phase CT abdomen was ordered, which showed non-visualization of the main portal vein with portal cavernoma causing minimal central IHBRD with a sign of portal biliopathy, as shown in Figure 1.

**Figure 2:**
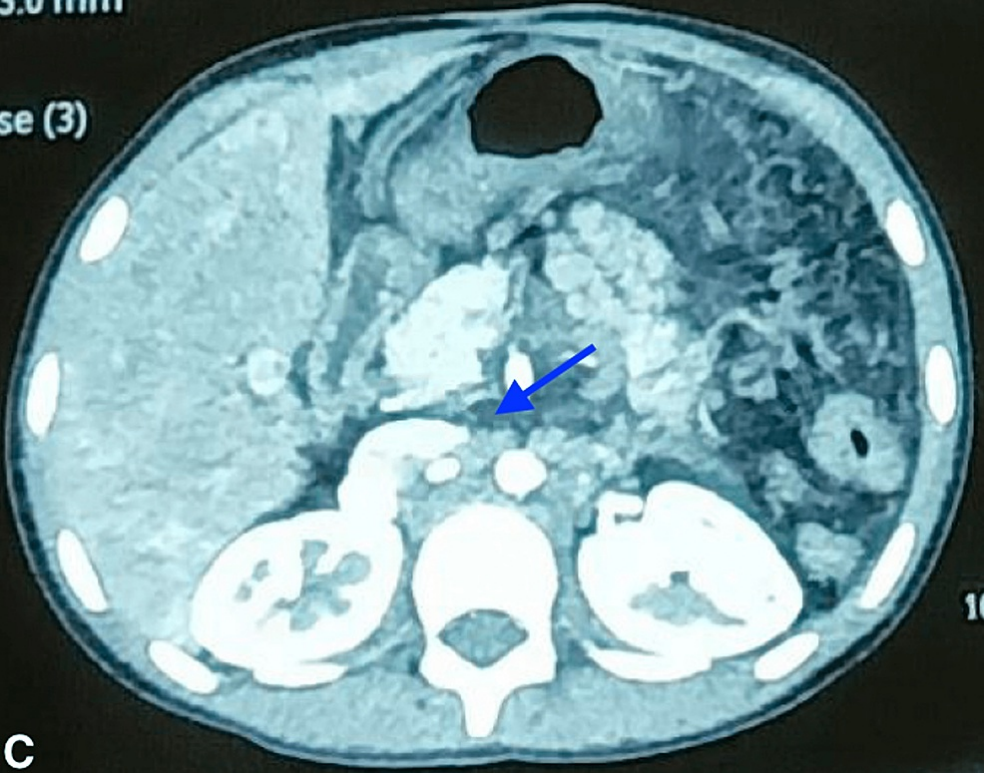
Multiple hypodense sub-centimetric mesenteric and retroperitoneal lymph nodes seen

In addition, the surgically created splenorenal shunt was not well visualized, and multiple hypodense sub-centimetric mesenteric and retroperitoneal lymph nodes likely secondary to mesenteric congestion were noted, as shown in Figure 2.

Because of the foregoing findings, informed consent was obtained for fresh shunt surgery, and the procedure went smoothly. The patient was discharged and followed up on an antibiotic, multivitamin, and ecstasy regimen.

## Discussion

As much as 30% of cases of upper GI hemorrhage in children are deadly, with portal hypertension being the most common cause. The most frequent cause of pediatric portal hypertension is an EHPVO. It is unclear what causes portal venous obstruction and which factors increase the risk of upper GI bleeding in children with EHPVO. Most bleeding episodes are spontaneous, while some may be brought on by drug consumption or a feverish condition. In EHPVO, the portal vein is typically blocked, with extensions into the splenic vein and occasionally the superior mesenteric vein. Only the portal vein's terminus at the hilum is blocked in tiny amounts. Sometimes, the entire splenoportal axis is blocked, although often, the blockage is at the portal vein development. Traditionally, the liver is normal, and the architectural pattern is preserved in EHPVO instances. Concentric condensation of reticulin fibers surrounding portal tracts may be seen histopathologically and sometimes reaching into the parenchyma. In EHPVO, there have been reports of altered hepatic storage capacity and transit maxima for bromsulphalein and lidocaine (monoethylglycinexylidide) excretion. A liver biopsy is typically unnecessary in EHPVO patients unless liver functions are dysregulated.

EHPVO is most often present in a child with splenomegaly, upper GI bleeding (expressed as either hematemesis or melena), and no signs of chronic liver disease. A child with portal hypertension and normal liver function, along with the lack of any virologic markers, are additional indicators of EHPVO. However, it is essential to rule out other causes of compensated childhood cirrhosis and non-cirrhotic portal hypertension. The basis for the diagnosis of EHPVO is imaging of the spleno-portal axis. EHPVO is diagnosed by Doppler ultrasound, CT scan, or MRI imaging, which shows portal vein obstruction, the presence of intraluminal material, or portal vein cavernoma, following the Baveno V consensus statement. Demonstrated portal venous blockage without cavernoma is a hallmark of recent EHPVO [5]. Additionally, used procedures include variceal obliteration and prophylactic endoscopic sclerotherapy (EST). Over a median follow-up of 4.5 years, children with portal hypertension who received prophylactic EST experienced fewer bleeding episodes (24%) compared to controls (42%) [6]. However, there are insufficient data or experience with youngsters to propose prophylactic EST or endoscopic variceal ligation (EVL). More evidence supports secondary prophylaxis than primary prophylaxis. Beta-blockers are used to lower portal pressure and the danger of variceal hemorrhage. The standard dosage for propranolol is 2 mg/kg body weight divided into two daily doses. The dosage is checked and adjusted to avoid a reduction in heart rate of more than 20% from the baseline or an absolute heart rate below 60 beats per minute. Beta-blockers are beneficial in preventing variceal bleeding, according to a meta-analysis [7].

The treatment for symptomatic patients with recent EHPVO should begin with oral anticoagulant therapy and low molecular weight heparin as soon as it is detected. Anticoagulation should be considered in asymptomatic patients if it is discovered early. Anticoagulation therapy dosage must be gradually increased to maintain an international normalized ratio (INR) of 2 to 3. The Baveno V consensus statement advises anticoagulation for at least three months unless long-term or lifelong therapy is necessary for an underlying persistent prothrombotic condition [5]. Shunting and non-shunting techniques are used in the surgical therapy of EHPVO. By moving blood from the high-pressure portal venous system to the systemic circuit, the shunt treatments work to lower portal pressure. Shunt operations are advised for EHPVO instances that do not respond to medicinal or endoscopic therapy or if there are problems. Failure of endotherapy to control bleeding, the presence of stomach or ectopic varices (not manageable by endoscopic procedures), and accompanying problems such as portal biliopathy and extensive rectal varices are all grounds for shunt surgery. Nowadays, emergency shunt surgeries are performed relatively infrequently because endoscopic care is so widely available. Our patient was not compliant with the follow-up due to limited resources, and eventually, shunt surgery was performed because of increasing complications.

## Conclusions

EHPVO is a common cause of portal hypertension without cirrhosis. Children exhibit different clinical presentations than adults. Obstruction of the portal vein leads to cavernomatous transformation that predisposes portal hypertension complicated in esophageal varices. Portal biliopathy, hypersplenism, ectopic varices, and growth retardation develop from the extensive collateral formation. Variceal bleeding in EHPVO is endoscopically managed by band ligation and sclerotherapy with low morbidity. Portosystemic shunt surgery shows a good response in bleeding prevention, hypersplenism, portal biliopathy, growth retardation, and complicated cases, as in this case.

## References

[1] Elkrief L, Houssel-Debry P, Ackermann O (2020). Portal cavernoma or chronic non cirrhotic extrahepatic portal vein obstruction. Clin Res Hepatol Gastroenterol.

[2] Khanna R, Sarin SK (2018). Idiopathic portal hypertension and extrahepatic portal venous obstruction. Hepatol Int.

[3] Zielsdorf S, Narayanan L, Kantymyr S (2021). Surgical shunts for extrahepatic portal vein obstruction in pediatric patients: a systematic review. HPB (Oxford).

[4] Zhang J, Li L (2022). Rex shunt for extra-hepatic portal venous obstruction in children. Children (Basel).

[5] de Franchis R (2010). Revising consensus in portal hypertension: report of the Baveno V consensus workshop on methodology of diagnosis and therapy in portal hypertension. J Hepatol.

[6] Goncalves ME, Cardoso SR, Maksoud JG (2000). Prophylactic sclerotherapy in children with esophageal varices: long-term results of a controlled prospective randomized trial. J Pediatr Surg.

[7] Sarin SK, Guptan RK, Jain AK, Sundaram KR (1996). A randomized controlled trial of endoscopic variceal band ligation for primary prophylaxis of variceal bleeding. Eur J Gastroenterol Hepatol.

